# Humming and Homeostasis: Insights from Infants, Mothers, Mantras and Caregiving

**DOI:** 10.3390/bs16050805

**Published:** 2026-05-18

**Authors:** Maya Gratier, Maria Eduarda Carvalho

**Affiliations:** 1 Laboratoire Ethologie Cognition Développement, Université Paris Nanterre, 92000 Nanterre, France; 2 CESEM-NOVA-FCSH, Universidade NOVA de Lisboa, 1099-085 Lisboa, Portugal

**Keywords:** humming, homeostasis, social interaction, interoception, vibration

## Abstract

Humming is a ubiquitous yet understudied form of human vocalisation that may play a role in regulating internal bodily states during early development. Unlike speech or singing with lyrics, humming consists of continuous, semantically unstructured vocal sounds produced with a closed mouth. It generates sustained sonic, vibratory and respiratory patterns that engage interoceptive and autonomic processes. This paper explores the hypothesis that humming critically contributes to homeostatic regulation in caregiver–infant interaction. Drawing on research in developmental psychology, perinatal care and vocal practice, we propose that humming provides a simple mechanism through which caregivers may scaffold the infant’s developing interoceptive awareness, paving the way to subsequent social cognition abilities. Through slow rhythmic breathing, chest and cranial vibration, and temporally structured vocal phrasing, humming may influence cardio-respiratory rhythms and support autonomic balance while also organising moments of social engagement. Evidence is gathered from studies of maternal humming during kangaroo care with preterm infants, showing that these vocalisations can stabilise physiological parameters and invite infant vocal participation. We argue that humming may function as an embodied, interoceptive form of co-regulation for both infants and caregivers, linking physiological homeostasis with early communicative exchange.

## 1. Introduction

Humming is a sound produced by the vibration of the vocal folds. Unlike speech, humming involves no rapid transitions between discrete sounds but rather continuously gliding pitch movement or rising and falling gradual pitch steps. Unlike singing, humming involves no semantic content and can be produced with a closed mouth while breathing through the nose (Snow et al., 2018; Stevanovic, 2013). Some humming sounds consist of entire or parts of recognisable melodies while others are spontaneous and improvisational with no clearly identifiable musical template. Humming is a unique type of vocal production, involving high resonance of the vocal cords with vibratory propagation through the vocal tract resonators, thorax and along the spinal cord. It results in vagus nerve stimulation and the production of nitric acid in the paranasal sinuses (Woo & Kim, 2025). It is usually organised around long sound-forming exhalations interspersed with slower air intake than in singing with lyrics (N. F. Bernardi et al., 2017). It is thus intimately connected with cardio-respiratory processes and autonomic regulation of internal states. All living organisms rely on homeostasis for their autonomic regulation (Cannon, 1929), that is, the ongoing regulation of bodily and physiological states within optimal ranges through feedback from the environment and from the body itself (Fotopoulou & Tsakiris, 2017). Recent models suggest that homeostasis works in conjunction with allostatic processes, involving not only reactive adjustments to environmental conditions but also predictive adaptations to fluctuations caused by changes in the environment (Atzil & Feldman Barrett, 2017; Diaz-Rojas et al., 2025). The types and qualities of events, particularly in the social environment, are central to these processes. Through a narrative literature review, this paper puts forth the idea that perinatal humming, because it affords interpersonal multisensory integration, is a powerful means for establishing and maintaining homeostasis for both caregivers and infants. Perinatal humming may not only support well-being for caregivers and infants but also further prepare infants for the developmental challenges that lie ahead.

## 2. Humming with Infants in Caregiving Contexts

In infancy, speech and song are known to play important roles in affect regulation through melodic and rhythmic sound shaping. Infants are born with intricate sound processing abilities and are cued to respond to the complex and changing acoustic features of the human voice. Parents’ and caregivers’ speech can at times resemble singing as reflected in the now well-known music-like characteristics of infant-directed speech (Papousek, 1996). Much research has focused on infant-directed speech, on the performances of musical babysongs and poetry-related nursery rhymes, showing that infants are exposed to an array of expressive human sounds in their daily lives (Politimou et al., 2018). Listening to speech and music starts in the womb and prepares the foetus for life in a world of human-made sound. Human sound-making can be meaningful and immediately useful such as in speech and singing, it can also be meaningless and seemingly gratuitous, such as in humming, toning or whistling and it can indicate urgent needs or internal physiological states such as in screaming, gasping, groaning, laughing or crying. In this paper, we focus on humming as an understudied form of vocal expression which, we suggest, plays an important role in infancy, including prenatal life.

Although the literature on humming in caregiving practices with infants is scarce, a few studies suggest that it engages infants’ physiological states, promotes co-regulation, and facilitates temporally structured vocal interaction from the earliest moments of postnatal life. This fits with broader theories of caregiver–infant coordination, where vocalisation has been shown to scaffold attention, regulate arousal and support social engagement (Trevarthen & Reddy, 2017).

The only studies conducted specifically on the effects of maternal humming on infants have focused on face-to-face, skin-to-skin kangaroo care with premature infants in the neonatal intensive care unit (NICU). Preterm infants show lower heart rate and higher oxygen saturation during maternal humming compared to silence or speech, suggesting infants spend more time in a state of homeostasis during a maternal humming condition (Carvalho & Justo, 2024). In addition, humming during kangaroo care was associated with higher ratios of oxygen saturation/heart rate in mothers and can thus be thought to improve maternal cardio-respiratory function as well. Furthermore, certain acoustic features of maternal humming, such as type of melodic contour and phrase-final lengthening, predicted greater physiological stability in preterm newborns (Carvalho & Justo, 2024). More specifically, phrase-like vocalisations with greater pitch variation and a marked lengthening of the final note were associated with a higher oxygen saturation/HR ratio in preterm infants (Carvalho & Justo, 2024). Prolonged pitch modulation and phrase-final lengthening are central features of lullaby style infant-directed singing, a musical form whose function it is to regulate and soothe infants’ states of awareness.

Surprisingly, in this context, preterm infants were found to vocalise more frequently on the phrase endings of maternal humming (Carvalho et al., 2020). Acoustic analysis of maternal humming suggests that the in-breath that follows the lengthening of the final note may be considered a physiological marker of the offset pauses between vocalisation units. We think that this marker may afford the infants’ vocal responses at the end of the final note, suggesting that preterm infants can predict the end of hummed phrases. Another study of maternal singing during kangaroo care in the NICU showed that it induces communicative behaviours in both mothers and infants compared to kangarooing without singing (Provasi et al., 2021) and suggests that singing with skin-to-skin contact creates, for the premature newborn, the kind of interconnected rhythmic experiences that are characteristic of prenatal life.

Additional evidence of the effects of humming on newborns is provided by The Singing Kangaroo project, a Swedish–Finnish multi-centre randomised control trial on the impact of parental singing during kangaroo care in the NICU (Hugoson et al., 2025). Frequent parental singing, including humming, over a period of 4 weeks was found to enhance auditory discrimination of speech sounds, with correlated neural responses measured with EEG (Kostilainen et al., 2021; Partanen et al., 2022). Studies also show important benefits of spontaneous singing on parental well-being, self-esteem and sensitivity to infant signals (Haslbeck, 2014; Haslbeck et al., 2021). Singing was found to promote affective bonding between parents and infants (Hugoson et al., 2025). More generally, a growing body of evidence on infant-directed vocal communication with both premature and full-term infants points to the crucial role of vocal contact for social interaction, cognitive development and brain maturation (Filippa et al., 2017, 2020; Haslbeck, 2012; Standley, 2012).

Such research findings corroborate the practice of relationship-based music therapy in the NICU setting. In Creative Music Therapy (CMT), for example, the therapist hums or sings in an infant-directed lullaby style. The hummed song is continually adjusted to the body movements, emotional expressions and breathing patterns of the preterm infant (Haslbeck et al., 2017, 2023). CMT can be practiced with newborns in their incubators when they are in a stable and relaxed state. It is effective for soothing or stimulating premature infants in a co-regulated manner, at a time when many other types of interventions are still risky and can overwhelm this highly vulnerable patient group. Another form of CMT can be used during kangaroo care where the music therapist accompanies parental vocal humming with a monochord instrument. Based on the principles of family-integrated care, parents are thus included in the therapeutic process, delivering CMT during kangaroo care (KC) and engaging in vocal interaction with their infant. The principles of CMT are closely related to the spontaneous “communicative musicality” of vocal interaction between parents and young infants (Malloch & Trevarthen, 2009), describing the matching qualities and rhythms of parents’ and infants’ vocalisations. For example, in CMT, the therapist will match vocal pitch and tempo to the upward movement of an infant’s eyebrows with an upward pitch glide and increasing tempo. Conversely, if the infant is overly aroused, the therapist will broaden the melody’s pitch range and shift it downwards while slowing the tempo as well as repeating and prolonging the phrase-final notes. Another interesting feature of the CMT method is the attunement to environmental sounds, such as monitor beeps, during the improvisational singing, thus incorporating them into the tapestry of musical sound and thereby mitigating their disturbing effects (Haslbeck & Bassler, 2020).

Even though parental humming, as a distinct form of parental vocal communication, does not appear to have been studied in full-term infants, research on live infant-directed singing offers useful insights for future research on humming specifically. Lullabies are a ubiquitous, age-old form of live singing aimed at soothing infants and regulating their states of awareness toward sleep (Mehr & Krasnow, 2017; Trehub, 2003). Humming has been described as a central feature of lullabies across cultures (Aubinet, 2024). Live singing, which is expressive, improvisational and attuned to infants’ changing states of awareness and emotion, has been found to prolong infant attention and regulate emotional states (de l’Etoile, 2006; Nguyen et al., 2023). Hummed lullabies may be particularly soothing for infants for offering highly predictable sound experience corresponding to the rates of respiration of the singer and closely matched to those of the infant. They may scaffold a gradual slowing of cardio-respiratory variability during the infant’s transition to a sleep state. Furthermore, in this context, lullaby humming may indeed play an important role in regulating the caregiver’s own internal states, as shown in the study of Carvalho and Justo (2024) with premature infants.

Humming is thus a relevant caregiving practice not only for further supporting caregiver–infant bonding during kangaroo care but also for the development of physiological co-regulation throughout infancy. Humming in a lullaby style with calm, slow, simple, predictable, and repetitive vocalisations may optimise the cardio-respiratory functioning of both the caregiver and the infant.

## 3. Well-Being and the Physiology of Group Singing

Voice production relies on a complex array of dynamically organised anatomical and physiological subsystems, involving feedback and feedforward processes. It relies on large numbers of muscles, facial, spinal and cranial nerves, subcortical and cortical parts of the brain and cardio-respiratory processes distributed across the central and autonomous nervous systems. Importantly, voicing mobilises and integrates visceral, pulmonary and neural control mechanisms (Zamorano et al., 2025). Because respiration is a driving force of voice production and control, it is intimately linked to the equilibrating processes involved in homeostasis. The ways in which singing affects the body and hence psychological affective states pertaining to stress, sociality and well-being, has recently come under greater scrutiny. Humming and chanting have been found to lead to a reduction in blood pressure, heart rate, and breathing rate (Lehrer & Gevirtz, 2014).

The benefits of group singing in particular are well-documented. Research has shown, for example, that the practice of collective singing enhances feelings of belonging and group cohesion (Welch et al., 2014) and reduces individual heart rate variability (HRV) (Vickhoff et al., 2013). Cooperative non-face-to-face humming with another person was shown to increase interpersonal synchronisation of HRV (Vickhoff et al., 2013) and inter-brain neural synchrony (Osaka et al., 2014). Such findings have encouraged the use of choral singing in a widening array of therapeutic, rehabilitation and educational contexts (Clift & Hancox, 2001; Stegemöller et al., 2017).

Yet the relation between low-level physiological aspects and higher-order effects of such practices are not sufficiently well-understood. Singing in groups involves numerous higher-order goals and processes including sound production and feedback, agency and self-control, aesthetic motives, social coordination and emotional contagion (Hao et al., 2026). Multiple physiological correlates are involved, such as cardio-respiratory function, and oxytocin and cortisol regulation, but it is not easy to know which might be driving or constraining experiences of well-being associated with group singing.

For example, compared to freely humming, a continuous tone or singing a hymn with piano accompaniment, slow mantra chanting in groups is associated with a greater increase in HRV, which is a predictor of cardiovascular health, and higher HRV synchronisation between singers (Vickhoff et al., 2013). Musical and linguistic structure is thus important for guiding respiratory control and HRV. Shared structure has the potential to organise and regulate experiences within groups engaged in a common vocal practice. Meditative and ritual vocal chanting in various cultures is associated with an average regular breathing frequency of 0.1 Hz (L. Bernardi et al., 2001a), which is optimal for cardiovascular function (L. Bernardi et al., 2001b) and points to a biological, common-sense grounding for many spiritual or healing practices around the world. It has been suggested that positive psychological outcomes are related to this tempo of six breaths per cycle, a breathing rate that promotes greater relaxation. The physiological demands of chanting thus play a causal role in reducing stress through increased parasympathetic activity.

However, in most studies of group singing or chanting, the effects of individual sound-guided respiration rates on HRV and those of rhythmic and motor entrainment between singers are confounded. Moreover, it is difficult to know whether it is regular breathing or the repetitive sound itself that affects HRV. Finally, the form and style of singing (predetermined structure vs. free improvisation) could also modify the relationship between singing and HRV. Elucidating these issues is important for understanding the role of perinatal humming for infant well-being and development.

Humming has been found to increase nasal nitric oxide which improves blood oxygenation (Weitzberg & Lundberg, 2002). One study on the effects of individual humming in an improvisational manner (called toning in this study) found that it favoured a breathing rate of around 0.1 Hz, as in meditative chanting practice (N. F. Bernardi et al., 2017). Furthermore, this study showed that similar cardio-respiratory optimisation was associated with soundless breathing at the same rates, suggesting that breathing pattern is the key factor for cardiovascular function. Indeed, slow-paced breathing promotes autonomic stability by enhancing parasympathetic activity and reducing the experience of stress (Czub et al., 2024). It also appears to have a cascading effect on higher-order cognitive functions such as attention control at the individual level (Laborde et al., 2021). Humming may thus be considered a cost-effective practice for stress-reduction and cardiovascular health.

Studies of the effects of singing and humming on hormonal and neuropeptide activity provide clues to understanding the role of humming not only for individual well-being but also for interpersonal regulation. Raised levels of oxytocin have also been reported in relation to group singing practices and are associated with reported well-being (Grape et al., 2003; Kreutz, 2014). It is quite relevant that an increase in oxytocin in musicians is particularly linked to collective improvisational practice involving vocal coordination and prosocial interaction (Keeler et al., 2015). Furthermore, the intranasal application of oxytocin has been shown to increase positive interactions between performers and reduce performance anxiety (Sabino et al., 2020). Overall, the literature points to a trend for increased endogenous oxytocin and reduced cortisol in subjects involved in musical activities, particularly spontaneous community music-making (Bowling et al., 2022; Busse et al., 2026; Good & Russo, 2022). It seems then that musicality, cooperative prosocial interactions and the oxytocinergic system are linked to neural activity in several common regions and interconnected networks in the brain (Harvey, 2020).

Much is known today about the link between oxytocin and caregiving in infancy. Marked increases in oxytocin are found in both parents and infants during affectionate play and nurturing interactions (Feldman, 2012b; Strathearn et al., 2012). Can musical caregiving be considered a developmentally relevant practice that affects both infants’ and caregivers’ core motivational and regulatory systems?

## 4. Interoception and Homeostasis in Infant Development

At birth, infants have limited autonomic flexibility and immature cortical control over arousal (McNamara et al., 1998). Independent regulation of body temperature, feeding cycles or stress is challenging for very young infants. Many of their homeostatic systems are functional but only become self-stabilised through participation in a broader regulating system, one that includes caregivers and caregiving practices tuned to their needs (Durán & Isaac, 2026). Importantly, it is the coupling between an infant’s internal states and their perception and transformation by caregivers that provides the infant with growing expertise in assessing and controlling their own internal processes. Attuned social caregiving both regulates homeostasis and supports the development of allostatic regulation toward an independent allostasis system (Diaz-Rojas et al., 2025). Allostasis is the continual predictive adaption of one’s internal state to a changing environment, whereas homeostasis is a physiological process that restores internal imbalance.

During everyday, recurrent social and caregiving interaction, infants achieve states of physiological equilibrium through such activities as feeding (which regulates glucose), holding (which regulates temperature, heart rate and cortisol), or vocal speech or singing (which regulate respiration, vagal tone and oxytocin). Repeated daily experiences of losing and regaining physiological stability through sensitive caregiving provides infant brains with dynamic roadmaps for mapping their internal bodily states. Infants learn the pathways towards states of homeostasis associated with well-being and pleasurable experience with caregivers. Thus, homeostasis-oriented social interaction shapes infants’ interoceptive prediction models and supports allostasis (Durán & Isaac, 2026). Interoception refers to the nervous system’s capacity to sense, perceive, interpret, and integrate signals originating from within the body (Craig, 2002). It has been shown to be crucial for maintaining physiological homeostasis (Critchley & Garfinkel, 2017).

A substantial amount of research on caregiver–infant interaction has shown that it mediates the development of infants’ sense of bodily self, awareness of self and others, and eventually social cognition (Feldman, 2012a; Porges et al., 1994; Trevarthen, 1993). It has also paved the way for more recent work showing the connections between caregivers’ own interoceptive sensitivity and their capacity to attune and synchronise behaviourally with young infants through multisensory channels such as voice and touch (Ciaunica & Crucianelli, 2019; Montirosso & McGlone, 2020). The specific processes through which very young infants and caregivers together negotiate allostasis, the continual predictive adaption of their own internal state to a changing environment (Diaz-Rojas et al., 2025), requires more attention. Recent neuroscientific evidence from animal studies and human adults suggests there are two modes for processing interoceptive signalling: an information mode supporting bodily regulation and a coordination mode underlying unified conscious or subjective experience (Tallon-Baudry et al., 2026). Does attuned interpersonal interaction, supported by musical practice such as humming, support the development of interoceptive awareness in both infants and caregivers? Knowledge of interoceptive processing during multisensory interaction may provide insight into the development of homeostasis and allostasis, which themselves are fundamental for social and cognitive development.

## 5. Skin-to-Skin Humming as Co-Regulation of Infant and Caregiver

Singing is a deeply embodied activity which has been found to be associated with enhanced interoceptive awareness (Zamorano et al., 2025), supporting the integration of both internal and external bodily inputs. Could a situation such as perinatal humming, where the caregiver produces repeated and varied nonverbal vocal sound that the infant perceives both aurally and as vibrotactile input, enhance the infant’s interoceptive awareness?

Recent studies of biobehavioural synchrony in parent–infant interaction reveal processes of co-regulation of respiration rates resulting in cardio-respiratory synchrony (DePasquale, 2020; McFarland et al., 2020; Miller et al., 2023). Van Puyvelde et al. (2015) studied mother–infant physiological relatedness longitudinally after birth by positioning infants on their mothers’ body with skin-to-skin contact and instructing mothers to breathe at different rates. Until 2 months, infants were found to adjust their respiratory sinus arrythmia (RSA) rates to their mothers’ suggesting a strong dependency on the mother’s body for homeostatic regulation during the first 2 months of postnatal life.

As described earlier, humming, both in face-to-face distal and in skin-to-skin proximal interaction between caregiver and infant, is associated with increased HRV in both partners and synchronised cardio-respiratory functioning (RSA). Increased HRV, if it remains within the normal range, is believed to reflect a greater capacity of the body to dynamically adjust to continually changing internal and external environments, and to reflect a healthy interplay between the sympathetic and parasympathetic branches of the autonomic nervous system (Thayer et al., 2010). Much research has shown how RSA relates to social behaviour in the parent–infant dyad (Feldman, 2006). Caregivers have an influence on RSA maturation (Field et al., 1995; Schore, 2015), which shows marked increases between 4 and 10 months of age.

In infancy, social engagement involves discontinuous attention to the other person. Infants’ attention cycles between visual orientation and withdrawal, as was shown long ago in a seminal paper by Brazelton et al. (1974). Some studies have suggested that so-called social withdrawal can be explained by attention to inner bodily states and processes. Within the “mentalising homeostasis” framework proposed by Fotopoulou and Tsakiris (2017), ongoing sensory experience both from within the body and from the environment, which are closely tied, involves a dynamic updating of neural models tracking homeostasis in the body. Interoception, according to this view, is shaped by early social interaction, especially through social touch in caregiving contexts.

More research is needed on the components of humming and the specific ways in which it affects infants’ developing autonomic nervous system through the dynamic interplay between homeostasis, interoception and allostasis. The first hypothesis worth testing is that humming with infants constitutes a low-demand co-regulation framework, both for infants to experience interoception and to learn about their bodily states and feelings, and for caregivers’ own regulation of stress. In particular, the multisensory integration of sound and vibrotactile experience associated with skin-to-skin caregiver humming, where vocal sound propagates through bone conductance and spinal cord receptors as well as auditory pathways, may be a powerful conduit for the development of an embodied sense of self in infancy.

## 6. The Power of Song in Prenatal Life and Labour

Although the postnatal months are crucial for ANS-related development of homeostatic control and interoceptive prediction, by the third trimester of gestation, key experience-expectant elements of the nervous, endocrine and immune systems of the foetus are already developed and functioning for the processing of affective states (Novak, 2004). Furthermore, the mother’s neuroendocrine condition (such as in response to stress or as a reaction to something pleasant or relaxing) produces related reactions in the foetus (DiPietro et al., 2003). Environmental sounds, such as unpleasant urban noise or the pleasant sounds of music or nature, affect the foetus in multiple ways. They are directly perceived through a functional and complex auditory system which is selective, spatialised and predictive (Philbin, 2017). However, unpleasant features of sound such as excessive loudness or reverberation are filtered not by the foetus’ own auditory system but by the medium within which it lives, that is the mother’s body with its tissues and fluids. Animal studies have shown that sounds, both pleasant and unpleasant, also indirectly affect the developing foetus during the last trimester of gestation, by affecting the mother’s neuroendocrine and physiological state (Barzegar et al., 2015). Loud, unpleasant, stressful sounds provoke surges in maternal cortisol, which in turn provoke increased cortisol in the foetus’ blood (Gélat et al., 2019). Noise pollution is clearly a major public health risk for pregnant women and infants.

Strong evidence of foetal auditory perception and learning is provided by a growing body of foetal neuroscience research. Auditory experience, mediated by the mother’s voice as well as through exposure to external sound, tunes neural circuits to speech and music processing. The prenatal brain thus forms memory traces from repeated auditory experiences, such as speech sounds and sung melodies, that persist after birth (Partanen et al., 2013, 2022). Recent evidence suggests that the foetal brain does not simply detect sound but, in the last trimester, is equipped with integrated networks for processing the temporal structure of sound (Draganova et al., 2018).

Sounds produced by the mother dominate the soundscapes inside the womb. The most nurturing intrauterine vibroacoustic experiences are those provided by the deep regular rhythms of the maternal heartbeat and by the mother’s voice perceived as melody and prosody (DeCasper & Fifer, 1980; Moon et al., 2013; Nazzi et al., 1998). Melodies are particularly salient in utero, those produced by the mother’s own vocal expressions as well as those of other people’s speech and music played in her vicinity (Granier-Deferre et al., 2011). Neonatal recognition of the familiar melodies from life in utero, including melodies that define a “mother tongue” (Mehler et al., 1978), contributes to the regulation of the infant’s affective states. Above all, the familiar voice of the mother, with its unique melodic signatures, is preferred over other voices and bootstraps visual memory for the mother’s newly discovered facial features (DeCasper & Fifer, 1980; Sai, 2005).

What does a foetus experience when the mother sings and when she hums? In recent years, research using high-resolution 4D ultrasound has revealed surprising correspondences between maternal vocalisation and foetal movement (Marx & Nagy, 2015; Reissland et al., 2014; Castiello et al., 2010) and systematic relations between foetal behavioural states and orientation responses to maternal voice (Voegtline et al., 2013).

Prenatal singing is an intentional practice that has various objectives and forms. Very few studies have focused on the effects of prenatal singing on the foetus or newborn. Only one study found that prenatal singing affects neonatal behavioural state regulation (Carvalho et al., 2025). More studies have shown its effects on pregnant women. These show similar effects as studies on group singing in non-pregnant adults, that is, reduced cortisol, increased oxytocin and cardio-respiratory synchronisation. Several studies highlight the benefits of singing during pregnancy, facilitating the release of oxytocin and the reduction in cortisol (Wulff et al., 2021). They also show that women enrolled in prenatal singing classes experience less anxiety and depression, and present higher perceived closeness to the child and more secure attachment through to the postnatal period (Fancourt & Perkins, 2018; Persico et al., 2017; Van Willenswaard et al., 2017; Wulff et al., 2021). The use of vocal toning and humming has shown benefits for relaxation throughout pregnancy (Pierce, 1998).

Such findings highlight the relevance of singing and humming during labour. Indeed, the release of oxytocin during could be supported by vocalisation practices during labour (Pereira et al., 2025a, 2025b). Humming in a low tone has potential mental and physical benefits for women in labour and has been used by women across the world as a pain control strategy. Intentional vocalisation in labour is the production of sonified toned exhalations with an open mouth. The vibration that goes through the body during such toned humming can help women cope with and regulate pain during labour as well as focus the tremendous effort and energy involved in childbirth. Studies have found advantages in opening the glottis by vocalising during contractions in the second stage of labour, with a protective effect against perineal trauma, maternal and foetal distress and a reduction in the duration of labour (Pereira et al., 2024).

## 7. Humming and Vibration

Humming with a closed mouth generates a different vibroacoustic experience, for the person humming and for others in their vicinity, than humming with an open mouth. With the mouth closed, sound and vibration propagate and resonate throughout the body and along the skeleton (Chen et al., 2014). Forms of vocal humming are in fact part of ancient practices around the world and often used in ritual and spiritual contexts. Such practices have long been cast aside as ”esoteric”, “irrational”, “primitive”, “dubious” or even “dangerous”, but scientists have recently begun to study their role for health and well-being. Humming continues to be practiced daily in many cultures, both by individuals and collectively, in religious chanting, shamanistic rituals or mantra recitation. It is a core aspect of yoga and pranayama practiced not only across the Indian subcontinent for at least a few millennia but also increasingly within the globalised world’s growing industry of wellness and personal development.

Bhramari Pranayama is a yogic technique involving vibratory closed mouth humming based on resonance in the nasal passages, mouth and throat. A recent literature review of 46 studies found that it has beneficial health effects by activating the parasympathetic nervous system and lowering the levels of stress, anxiety, blood pressure and SNS activity (Chetry et al., 2024). Recent studies (Trivedi et al., 2023a, 2023b) have shown that Bhramari Pranayama is more effective in reducing stress than physical activity, basing the effect on the induced elevation of parasympathetic activity. In particular, the prolonged vocalised expiration phrase offers opportunity for vibrational experience implicating not only auditory but also tactile components of the sounds.

Leppänen (2018) suggests that prenatal singing constitutes a unique vibroacoustic experience that potentially modulates maternal–foetal interaction. Vibration through prenatal singing creates a mother–foetus relational field, as it passes through the bone structures of mother and foetus as well as the fluids and tissues that bind them together. In this vibroacoustic experience, the foetus is not a passive entity receiving a signal but rather an active partner co-experiencing a field of sensation. The kind of embodied sensorimotor experience that vocal humming might enable could play a fundamental role in foetal brain development. The ongoing sensorimotor interaction loops that unite mother and foetus feed into the changing nervous system and its sensory and motor sub-systems, building functional neural circuits (Craighero, 2024). Ongoing movement–feedback cycles enable the developing brain to better predict and therefore learn to attend to a changing environment. The vibratory qualities of humming may then provide a distributed experience, which is at once auditory, tactile and motor, that uniquely structures the emerging brain.

Such prenatal distributed sensorimotor experience is likely to lay the groundwork for capacities that are critical to caregiver–infant interaction at birth and beyond. Newborns’ “communicative musicality” (Malloch & Trevarthen, 2009) may thus be shaped through tighter and tighter coupling of sound and movement in utero, that is, through both movements generated by the foetus itself and the movements and sounds generated by the mother. 

## 8. Conclusions

According to ethnomusicologist Joseph Jordania, humming played a vastly underestimated role in hominin evolution (Jordania, 2009). He suggests that humming is a pervasive form of vocalisation that occurs largely outside of awareness. Humming blurs the boundaries between human song, speech and the kind of contact communication found in many other animals. In contemporary humans, it is an expression of well-being, involving relaxed, friendly states and affording generative improvisational musical exploration. Song-like humming may indeed be related to the ongoing sounds of encouragement and agreement that support and scaffold verbal conversation, as when an interlocutor enunciates a “Hm” sound while listening to the speaker (Stevanovic, 2013). Humming may then be considered both as a signal of affiliation and social bonding and as a powerful means of physiological emotional regulation and embodied self-knowledge.

Humming has not been the focus of much empirical research, especially in the context of parenting and infant development, but recent empirical studies on humming in care and communication with premature infants have provided new scientific insights into this phenomenon. This paper offers a roadmap for exploring how it may contribute to early development both before and after birth and invites researchers to focus on the connection between distributed vibroacoustic experience, interoception and homeostasis. It also crucially proposes that skin-to-skin perinatal humming regulates both infants and caregivers within a bi-directional interdependent process. Finally, by situating the infant, and indeed even the foetus, not as a passive receiver of stimulation but as a participant in interaction, this paper suggests that humming not only facilitates homeostasis by reducing stress and improving well-being but also offers opportunities for learning.

## Data Availability

No new data were created or analyzed in this study.
